# Genetic Architectures of Myeloid Dysregulation in Severe COVID-19

**DOI:** 10.3390/v18060604

**Published:** 2026-05-26

**Authors:** Darya A. Kashatnikova, Alesya S. Gracheva, Artem N. Kuzovlev, Lyubov E. Salnikova

**Affiliations:** 1 Vavilov Institute of General Genetics, Russian Academy of Sciences, 119991 Moscow, Russia; daria_sv11@mail.ru (D.A.K.); palesa@yandex.ru (A.S.G.); 2 Lopukhin Federal Research and Clinical Center of Physical-Chemical Medicine of Federal Medical Biological Agency, 119435 Moscow, Russia; 3 Federal Research and Clinical Center of Intensive Care Medicine and Rehabilitology, 107031 Moscow, Russia; artem_kuzovlev@mail.ru

**Keywords:** COVID-19, host genetics, rare high-impact variants, myeloid dysregulation, thrombo-inflammation, whole-exome sequencing

## Abstract

**Background:** Dysregulated myeloid responses are central to severe COVID-19, but the contribution of host genetics to this “emergency myelopoiesis” is poorly understood. **Methods:** We performed whole-exome sequencing in 77 hospitalized COVID-19 patients to analyze the impact of the cumulative burden of rare, high-impact variants (qualifying variants, QVs) in hemopoietic and inflammatory gene sets on longitudinal leukocyte counts. Predictive models were validated using repeated internal cross-validation (1000 resamples) and external population-scale exome data (Genebass, *n* > 380,000). **Results:** The QV burden was a significant predictor of peak neutrophil and monocyte counts, independent of age, sex, and clinical severity. This association remained robust in a microbiologically confirmed “pure viral” subcohort (*n* = 54) and was stable across internal cross-validation resamples. External validation revealed an 11.2-fold enrichment of myeloid-associated genes within our candidate gene sets (*p* ≈ 2.2 × 10^−63^). Patients with a high QV burden exhibited significantly worse outcomes, including a four-fold increase in mortality (*p* = 0.00065), and a genetic profile linked to hyper-inflammation and thrombosis. **Conclusion:** These findings suggest that host genetic architecture may contribute to the magnitude of myeloid dysregulation in acute viral infection. Genetic stratification could identify patients predisposed to a hyperactive myeloid response, potentially guiding early, targeted immunomodulation to mitigate severe complications.

## 1. Introduction

The clinical course of COVID-19 is remarkably heterogeneous, ranging from asymptomatic infection to critical illness. Beyond primary respiratory failure, SARS-CoV-2 triggers a profound immune-inflammatory and thrombotic response, leading to multi-organ dysfunction. Hematological disturbances are a hallmark of this severe phenotype. While lymphopenia is a well-established prognostic marker, the massive expansion of the myeloid compartment, particularly neutrophilia and monocytosis, is increasingly recognized as a potential contributor to collateral tissue damage, cytokine storm, and immunothrombosis [1,2].

While an individual’s baseline hematological profile is known to be highly heritable, this is primarily understood from population studies of healthy individuals, which have identified common regulatory variants through array-based methods [3,4]. However, rare coding variants, typically captured by sequencing, are known to have effect sizes much larger than common variants, suggesting they may be critical factors in extreme phenotypes [5,6]. Despite this, the influence of rare host genetics on the dynamics of the hematological response during acute COVID-19 is not well understood. It is unknown whether the genetic factors that regulate baseline hemopoiesis also govern “emergency myelopoiesis”—the rapid mobilization of myeloid cells from the bone marrow during severe infection.

To address this, we leveraged whole-exome sequencing to investigate the role of rare, high-impact functional variants in shaping hematological trajectories. Our approach builds on the principle of “collapsing analysis,” which assesses the cumulative burden of multiple rare variants within a gene or a set of related genes [7]. We have previously demonstrated the utility of this method in linking the cumulative effects of such variants to severe COVID-19 phenotypes and anemia [8,9].

In this study, we hypothesized that the magnitude of the myeloid response in severe COVID-19 is associated with an underlying genetic burden in key biological processes. To test this, we defined a comprehensive gene space by selecting genes annotated with the Gene Ontology (GO) terms “Hemopoiesis” (GO:0030097), “Immune Response” (GO:0006955), and “Inflammatory Response” (GO:0006954). This selection was based on specific rationales: (i) Hemopoiesis: These genes govern the production and differentiation of blood cells, and variants here may impair the bone marrow’s ability to regulate output during stress [10,11,12]. (ii) Immune response: Genetic defects in pathogen recognition and clearance are central to a dysregulated response [13,14]. (iii) Inflammatory response: As a critical subset of the immune response, these genes are directly implicated in the systemic inflammatory response and tissue damage [15,16].

We aimed to quantify the contribution of rare functional variants to the hematological profiles of acutely ill COVID-19 patients. A central challenge in this analysis is that severe COVID-19 often manifests as a sepsis-like hyperinflammatory syndrome, making it difficult to distinguish the primary viral response from the host’s response to secondary bacterial superinfection [17,18]. To address this, we integrated granular electronic health record (EHR) data, including longitudinal microbiological results from respiratory and blood cultures, to definitively categorize patient subgroups. Furthermore, we accounted for the impact of systemic glucocorticoid therapy—a standard-of-care intervention known to significantly influence leukocyte dynamics [19,20]. By analyzing these refined clinical strata and utilizing population-scale external validation, we sought to identify the intrinsic host genetic determinants of the dysregulated myeloid response to SARS-CoV-2 and link these predispositions to clinical outcomes, including mortality and thrombo-inflammatory complications.

## 2. Materials and Methods

### 2.1. Participants

This study included 77 unrelated individuals of European ancestry (45 males, 32 females) with a confirmed diagnosis of SARS-CoV-2 infection (PCR-positive) and whole-exome sequencing (WES) data. Patients were recruited in 2020 from three Moscow-based clinical centers [8,9] prior to widespread vaccination. The participants were hospitalized at the Moscow Regional Scientific and Clinical Institute named after M.F. Vladimirsky (*n* = 16), the Moscow Clinical Center for Infectious Diseases “Voronovskoe” (*n* = 10), and the City Clinical Hospital of the Moscow Department of Health named after V.P. Demikhov (*n* = 51). These clinics were coded as clinic 1, clinic 2, and clinic 3. Patients were excluded if they had an incurable terminal disease, known pre-existing hematological disease (including but not limited to myelodysplastic syndromes, myeloproliferative neoplasms, and leukemias), primary or acquired immunodeficiency, prolonged use of corticosteroids, pregnancy, alcohol or drug abuse, HIV/AIDS, or other chronic systemic conditions/infections deemed likely to independently cause significant immune or myeloid dysregulation (e.g., systemic vasculitides, end-stage organ failure). All patient records were manually reviewed for such conditions. The diagnosis and severity of COVID-19 were determined according to the International Recommendations for the Prevention, Diagnosis and Treatment of Emerging Coronavirus Infection [21] and the Interim Guidelines for Prevention, Diagnosis and Treatment of COVID-19 published by the Russian Ministry of Health [22].

Complications were diagnosed according to established clinical, laboratory, and imaging criteria: pulmonary edema and cerebral edema (radiological imaging), pleural effusion (chest ultrasound or CT), pulmonary embolism (PE) (Geneva score ≥ 11 and/or D-dimer > 500 ng/mL, confirmed by imaging) [23], disseminated intravascular coagulation (DIC) (ISTH criteria [24]), acute kidney injury (KDIGO criteria [25]), acute respiratory distress syndrome (ARDS) (Berlin definition [26]), and sepsis (Surviving Sepsis Campaign 2021 guidelines [27]). The selection of these specific complications for genetic analysis was based on three key factors: (i) their inherent clinical severity, (ii) a prevalence threshold (observed in ≥5 patients) to ensure analytical robustness in our cohort, and (iii) their well-documented pathophysiological relevance to severe COVID-19. The study protocol was approved by the local Institutional Review Board (AE 2.1.18), and written informed consent was obtained from all participants or their legal representatives.

Superinfection was defined as secondary bacterial pneumonia developing ≥48 h after admission for RT-PCR-confirmed COVID-19 [28]. Patients were divided into two groups based on the presence of bacterial pneumonia: the superinfection group (*n* = 23), comprising patients with clinical and radiological signs of pneumonia and a positive bacterial culture from sputum, bronchoalveolar lavage (BAL), or blood; and the COVID-only group (*n* = 54), comprising patients with confirmed COVID-19 who had no positive bacterial culture (all respiratory and blood cultures were negative or showed no growth below the clinical significance threshold of <10^4^ CFU/mL). Cultures were obtained at admission, weekly thereafter, or at the time of suspected superinfection (sharp clinical worsening). A patient was assigned to the superinfection group only if at least one bacterial culture was positive.

Information on glucocorticoid (corticosteroid) administration was extracted from the electronic clinical records. According to national and international guidelines current at the time of admission, patients with severe COVID-19 received a standard course of systemic glucocorticoids (*n* = 34). The most common dosage was dexamethasone at 8–20 mg/day or prednisolone at 150 mg/day, administered intravenously for 3–6 days. Steroid use was recorded as a binary variable (yes/no), based on documented treatment records.

### 2.2. Whole-Exome Sequencing and Variant Calling

WES procedures were detailed previously [9]. Briefly, genomic DNA was extracted from blood, and libraries were prepared using either the Swift 2S^®^ Turbo with Twist HumanCoreExome enrichment (Twist Bioscience, South San Francisco, CA, USA) (Genomed, *n* = 41) or Illumina TruSeq DNA Exome Kit (Illumina, Inc., San Diego, CA, USA) (Bio-bank Center, *n* = 36). Sequencing was performed on Illumina platforms (HiSeq X Ten, 2500, or 4000; Illumina, Inc., San Diego, CA, USA). Reads were aligned to the GRCh38 genome using BWA MEM (version 0.7.17) [29], and variant calling was performed with GATK HaplotypeCaller (version 4.6.2.0). Analysis was restricted to intersecting genomic regions. Variants were filtered based on GATK VQSR standards, additional quality metrics [8], and a minimum coverage of 10×. The mean sequencing depth was 76.74 ± 56.63.

### 2.3. Annotation of Variants

Variants were annotated using AnnoVar (ANNOVAR web version, accessed on 17 June 2024; https://annovar.openbioinformatics.org/en/latest/) and VEP (https://www.ensembl.org/Tools/VEP, release 112, accessed on 11 September 2024). Population frequencies were obtained from GnomAD v2.1.1. We focused on rare variants (Alternative Allele Frequency <0.001 or absent in GnomAD) found in ≤4 alleles within our cohort. Variants predicted to have a severe impact on protein function (e.g., stop-gain/loss, frameshift, splice site) were classified as high-impact (HI). We additionally assessed rare HI variants using CADD v1.7 PHRED scores, which predict the likelihood that a variant is harmful across the entire genome. A score of 20 or higher indicates that the variant is among the top 1% of all single-nucleotide variants (SNVs).

### 2.4. Gene Sets for Immune Cell Functions

Gene sets were compiled using the AmiGO 2 platform by querying GO terms: “immune response,” “hemopoiesis,” “inflammatory response,” “lymphocyte,” “monocyte,” and “neutrophil” (https://amigo.geneontology.org/amigo; accessed 18 June 2025). Functional annotation was performed using Enrichr [30] with GO Biological Process 2025 and Reactome Pathways 2024 modules to confirm relevant biological process enrichment.

### 2.5. Statistical Analysis

Statistical analyses were conducted using R (version 4.5.2). Primary analyses utilized core functions from the stats package, supplemented by the PCAtools, ggplot2, lmtest, caret, and broom.mixed packages for specialized tasks. Continuous variables were assessed for normality using the Shapiro–Wilk test. Between-group comparisons were performed using the independent samples T-test or one-way ANOVA for normally distributed data, and the Mann–Whitney U test or Kruskal–Wallis test for non-normally distributed data. Categorical variables were analyzed using the Chi-square test or Fisher’s exact test, as appropriate. A False Discovery Rate (FDR)-adjusted *p*-value of <0.05 was considered statistically significant for all analyses.

To assess the impact of genetic burden on white blood cell parameters, we employed Multiple Linear Regression (MLR). The genetic burden was defined as the total count of rare, predicted protein-disrupting qualifying variants (QVs) per individual, modeled under a dominant genetic model. Depending on the context of the analysis, all MLR models were adjusted for key covariates. The validity of each final MLR model was verified by checking the following assumptions: independence of residuals (Durbin-Watson test), homoscedasticity (Breusch–Pagan test), and absence of multicollinearity (all variance inflation factors, VIF, <5).

To control the false discovery rate (FDR) across all regression analyses, we applied a significance threshold of *p* ≤ 0.0151, corresponding to an FDR of 5% across all tested coefficients. This threshold was used for all model coefficients (with the exception of the intercept) to ensure the robustness of the reported associations across the full set of comparisons.

To mitigate the risk of model overfitting, a common concern in small-sample genetic studies, we adhered to the “one-in-ten” rule of thumb. We maintained a ratio of approximately 19 observations to 1 predictor (77 observations to 4 predictors: genetic burden, severity/outcome, age, and sex) in the whole cohort analysis. This ratio exceeds the recommended threshold of 10–15 observations per predictor required to ensure stable regression coefficients. In the subgroup analyses, there were no fewer than ten patients per variable.

To identify and control for potential technical batch effects, we performed Principal Component Analysis (PCA) on the matrix of aggregated genetic burden scores. The sequencing center and recruitment clinic were included as additional covariates in all post-hoc MLR analyses to ensure robust and confounder-adjusted effect estimates.

To evaluate the stability of the associations, we performed internal repeated 5-fold cross-validation (200 repeats, 1000 resamples) for all significant myeloid outcomes. For each endpoint, we compared a base model containing only clinical and demographic covariates (severity or outcome, age, sex) with a full model that additionally included the qualifying variant burden in either inflammatory response or hemopoiesis gene sets.

External validation was performed using a two-step approach. First, we used Genebass (https://genebass.org/ (accessed on 12 May 2026)), a resource of exome-based association statistics from UK Biobank participants. Genebass tests associations of rare coding variants with over 4500 human phenotypes using >380,000 UK Biobank exomes [31]. We queried the following phenotypes: monocyte count and percentage (phenotype codes 30130, *n* = 382,765; and 30190, *n* = 382,770), neutrophil count and percentage (phenotype codes 30140, *n* = 382,765; and 30200, *n* = 382,770). We included both absolute counts (cells/μL) and percentages (%) to ensure robustness. In COVID-19, myeloid expansion can be absolute (increased production) or relative (due to lymphoid depletion) [32]. Validating both ensures that the genetic signal relates to the fundamental regulation of myeloid cell proportion and abundance. Significant SKAT-O and/or burden test results (*p* < 0.05) for loss-of-function (LoF; i.e., high-impact) and missense variants at the gene level were aligned with our inflammatory response and hemopoiesis gene lists that contained QVs in our sample. Burden tests are powerful when most variants in a gene affect the trait in the same direction. SKAT-O (the optimal unified test) is superior when variants have different directions of effect or when only a small fraction of variants are functional. Using both allows for maximum sensitivity in identifying genes with diverse mutational architectures. While LoF variants provide the clearest evidence of functional impact, they are extremely rare. Including missense variants (often predicted to be damaging) captures a broader spectrum of the “mutational load” that influences complex traits like leukocyte counts.

As a second step of external validation, we performed an intensive literature search to support the functional evidence of our genes of interest with neutrophil or monocyte biology, stress-induced hemopoiesis, or related inflammatory pathways using the following query template in PubMed: ([Gene symbol] OR “Gene Name” [All Fields]) AND (“sepsis” [MeSH Terms] OR “COVID-19” [MeSH Terms] OR “inflammation” [MeSH Terms]) AND (“neutrophils” [MeSH Terms] OR “monocytes” [MeSH Terms] OR “myelopoiesis” [MeSH Terms]) NOT (“neoplasms” [MeSH Terms]).

### 2.6. Data Visualization

Figures were generated using R packages ggplot2 and ggfortify, the online tool SRplot [33], and Cytoscape (v3.10.3) [34].

## 3. Results

### 3.1. Clinical and Demographic Characteristics

**Figure 1:**
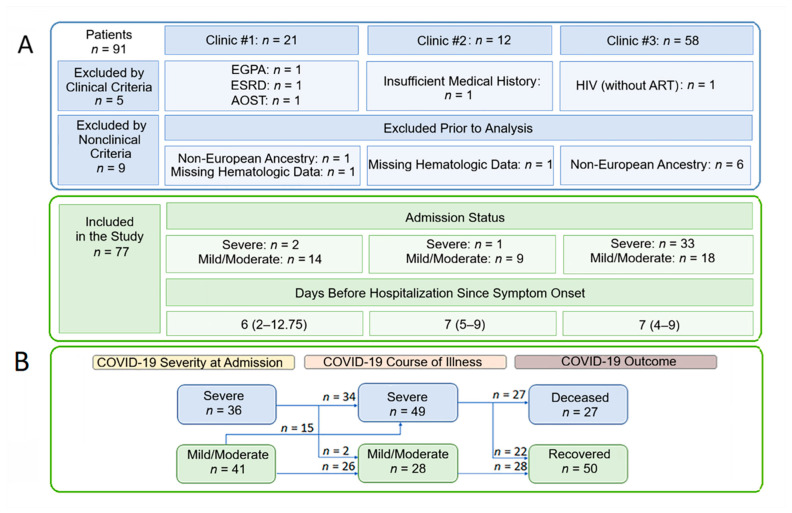
Patients eligible for the current study. (**A**) Patient selection flow diagram. (**B**) Schematic of patient distribution based on COVID-19 severity at admission, disease course, and outcome. The time from symptom onset to hospitalization is indicated by the median and the interquartile range (Q1–Q3). Abbreviations: EGPA, Eosinophilic Granulomatosis with Polyangiitis; ESRD, End-Stage Renal Disease; AOSD, Adult-onset Still’s disease; HIV, Human Immunodeficiency Virus; ART, Antiretroviral Therapy.

Following an initial screening of 91 patients from three clinics, five additional patients were excluded after manual review revealed complex comorbid conditions (EGPA, ESRD, Adult-onset Still’s disease, untreated HIV, or insufficient records) that could confound the analysis of COVID-19-specific myeloid responses (Figure 1A). For the genetic analysis, a further nine patients were excluded after verification of European ancestry through PCA-based clustering [8], and the availability of longitudinal hematological measurements, resulting in a final analytical cohort of 77 individuals. The cohort was stratified by clinical outcome and disease severity, assessed both at admission and across the disease course (Figure 1B; Supplementary Table S1).

Severe disease was defined according to WHO guidelines by the presence of SpO_2_ < 93%, respiratory rate > 30/min, or PaO_2_/FiO_2_ < 300 mmHg [21]. Patients who initially presented with severe disease largely remained in the severe category throughout the acute phase, with only two individuals transitioning to a non-severe classification. All fatalities occurred within the severe COVID-19 group. While demographics were similar across strata, comorbidities varied (Supplementary Table S1). The groups differed most significantly in ICU admission rates, mechanical ventilation requirements, and the extent of pulmonary lesions on CT. Complications were frequent, particularly AKI, ARDS, sepsis, and various forms of edema. Notably, ARDS and sepsis/septic shock significantly distinguished severe from mild courses, while pulmonary edema, cerebral edema, pulmonary embolism, and sepsis distinguished non-survivors from survivors. Bacterial superinfection (total *n* = 23/77) was significantly more common among deceased patients compared to those who survived (59.26% vs. 14%). Corticosteroids were administered to the majority of severely ill patients (34/49), including all 14 patients who were on invasive ventilation.

### 3.2. Blood Endotype Profiles and Longitudinal Trajectories

**Figure 2:**
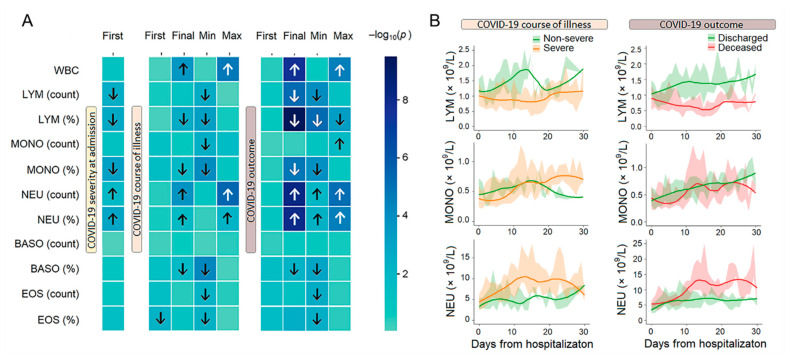
Blood endotypes and longitudinal patterns. (**A**) Differences in WBC parameters between groups (–log_10_ *p*-value, Mann–Whitney U test); arrows indicate the direction of significant changes (FDR corrected). (**B**) Longitudinal trajectories of the LEU, MONO, and NEU subpopulations in groups of patients stratified by severity and outcome over time. Data are presented as median values (solid lines) with interquartile ranges (IQR; shaded areas). Smoothed trends were estimated using LOESS regression. Analyses were restricted to the first 30 days of observation. Colors indicate clinical groups. Abbreviations: LYM, lymphocytes; MONO, monocytes; NEU, neutrophils; BASO, basophils; EOS, eosinophils.

We analyzed 11 WBC parameters using admission, final, and extreme (“min-max”) values (Supplementary Tables S2 and S3). At admission, severe patients exhibited significant imbalances in lymphocyte, neutrophil, and monocyte profiles (Figure 2A). When stratified by COVID-19 course, patients who developed a severe disease course showed a significantly greater increase in maximum WBC and neutrophil counts and percentages compared to patients with mild/moderate disease. In the “outcome” strata, the most pronounced differences were registered for both final and min–max measurements. The direction of these hematological changes was largely consistent with those observed at admission and during the disease course, with the notable exception of the max monocyte counts, which were significantly higher in deceased patients than in those who recovered.

Longitudinal tracking of immune cell profiles revealed clear patterns with a progressive divergence of trajectories during the observation period (Figure 2B). Beyond the absolute values of leukocyte counts, our analysis revealed that the kinetics of these cell populations were powerfully associated with disease severity and outcome (Supplementary Table S3). Specifically, we observed that in patients with severe or fatal COVID-19, the nadir of lymphocytopenia occurred significantly later and the peak of neutrophilia was dramatically delayed compared to non-severe or recovering patients. The divergent trajectories of lymphocytes and neutrophils in severe COVID-19 reveal a pathophysiological cascade. Severe disease is characterized not merely by lymphopenia and neutrophilia, but by a temporal uncoupling of their recovery: a failure to restore lymphocyte numbers precedes and likely enables a delayed, maladaptive surge in neutrophils (Supplementary Table S3).

### 3.3. Whole-Exome Summary Data

**Figure 3:**
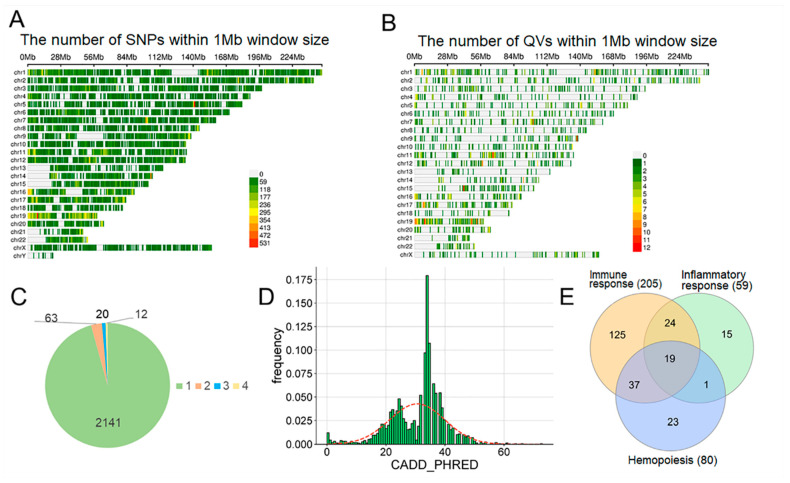
Genetic data overview. (**A**) SNP density per chromosome. (**B**) Qualifying variant (QV) density per chromosome. (**C**) Frequency of QVs in the patient sample. (**D**) Distribution of CADD PHRED scores of all QVs. (**E**) Venn diagram of genes containing QVs. Numbers in parentheses indicate the gene count.

Analysis of the COVID-19 patient cohort identified 105,294 unique genetic variants located within 19,409 distinct genes [9]. The chromosomal density distribution of these single-nucleotide polymorphisms (SNPs) is depicted in Figure 3A; observed gaps in the distribution correspond to known assembly gaps in the T2T-CHM13 human reference genome [35]. Among all identified variants, 3328 (3.16%) were classified as high-impact (HI). These are defined as variants predicted to be protein-disruptive, including splice acceptor, splice donor, stop-gained, frameshift, stop-lost, and start-lost variants. To enrich for potentially pathogenic variations, HI variants were filtered by rarity. Variants with a GnomAD alternative allele frequency (AF) below 0.001 or with no reported AF were termed ‘rare’. This subset of 2236 rare, high-impact variants was subsequently defined as qualifying variants (QVs) for downstream analysis. The genomic distribution of QVs (Figure 3B) was consistent with the overall SNP distribution.

Most QVs (95.75%) were singletons (Figure 3C), with a median CADD PHRED score of 30.5 (IQR: 23.35–34.00) (Figure 3D; Supplementary Table S4). We mapped QVs to three Gene Ontology (GO) sets: immune response, hemopoiesis, and inflammatory response (Figure 3E, Supplementary Figure S1). While the immune response set was the largest, the specific hemopoiesis and inflammatory sets overlapped significantly with it; only ~25–29% of genes were unique to these specific terms.

### 3.4. Effect of Genetic Variables on WBC-Related Parameters

**Figure 4:**
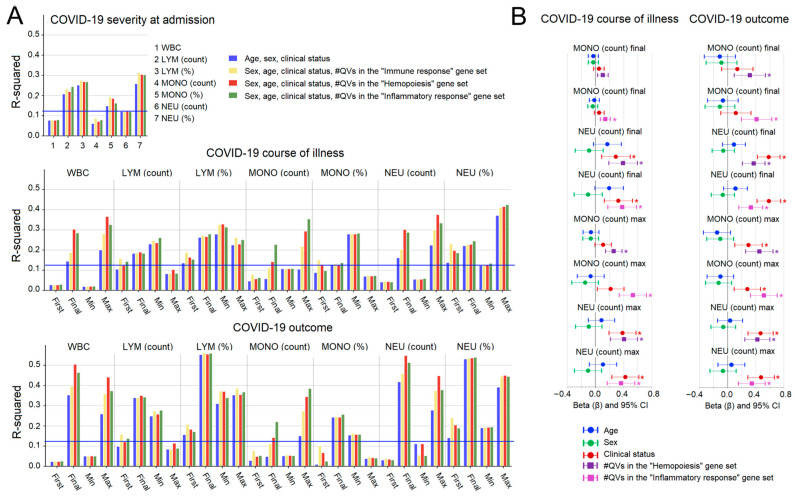
Multiple linear regression (MLR) analysis of genetic effects on WBC parameters. (**A**) Percentage of variance (R-squared) in WBC parameters explained by the full MLR model (clinical predictors + genetic predictor). Blue line: FDR-corrected significance threshold. (**B**) Standardized regression coefficients (beta) for the genetic predictor on monocyte and neutrophil counts. Covariates: age, sex, severity (left), and outcome (right). Asterisks (*) denote significant coefficients after FDR correction.

We used Multiple Linear Regression (MLR) to quantify the contribution of QVs to WBC parameters. Baseline models (age, sex, clinical condition) were expanded with the genetic burden (QV count) for each gene set (Figure 4A). Genetics did not significantly affect admission parameters, lymphocyte counts, or relative percentages (Supplementary Figure S2). Furthermore, any effects on basophil and eosinophil counts were minimal and largely non-significant (Supplementary Figure S2). However, genetics significantly predicted the final and maximum counts of total WBCs, monocytes, and neutrophils. Effect sizes were largest for the specific inflammatory and hemopoiesis gene sets. Comparing standardized coefficients (Figure 4B), the genetic contribution to final/maximum monocyte and neutrophil counts was comparable to or exceeded that of clinical condition, while age and sex were not significant predictors (Supplementary Table S5).

### 3.5. Multi-Level Validation of Signal Robustness and Biological Specificity

#### 3.5.1. Assessment of Technical Confounding and Batch Effects

We assessed potential technical artifacts arising from the use of different sequencing facilities and recruitment sites. Initial analysis revealed significant differences in genetic burden scores (PC1) between sequencing centers (*p* = 0.033) and recruitment clinics (*p* = 0.031) (Supplementary Figure S3). Further investigation showed that this “batch effect” was primarily driven by clinical heterogeneity: clinic #3 handled a significantly higher proportion of severe cases (82%) compared to clinics #1 and #2 (10% and 37%; *p* < 0.001), and these samples were sequenced exclusively at the Biobank Center.

Adjusting for these factors in multivariate models (Supplementary Table S5) showed that while sequencing center contributed to variance in some MLR models, the genetic burden (QV) coefficient remained independently significant. Recruitment clinic effects were non-significant after adjusting for severity, confirming the robustness of the genetic association.

#### 3.5.2. Stability of Genetic Associations Across Demographic and Clinical Strata

To ensure findings were not confounded by demographic variables, we first compared clinical myeloid counts across sex (males, *n* = 45; females, *n* = 32) and age subgroups (≤60 years, *n* = 38; >60 years, *n* = 39). No statistically significant differences in maximum or final counts were observed between these strata (Supplementary Table S6). Subsequent multiple regression analysis within these subgroups confirmed the robustness of the genetic associations, with qualifying variants (QVs) consistently predicting myeloid escalation regardless of age or sex (Supplementary Figure S4).

**Figure 5:**
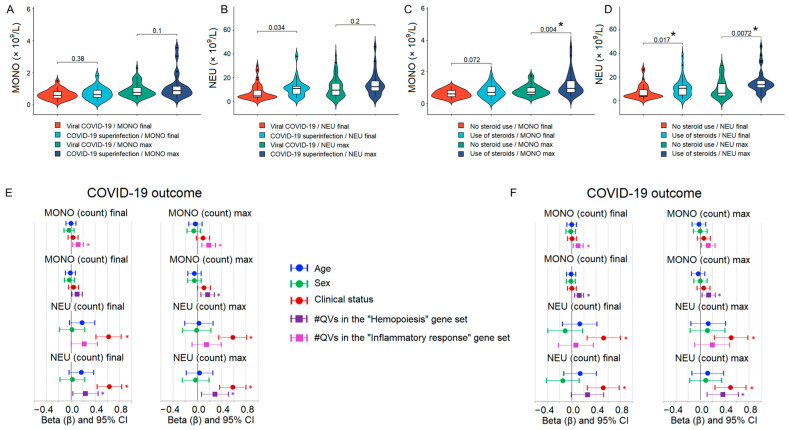
Comparison of neutrophil and monocyte counts between patient clinical subgroups and the results of multiple linear regression analysis. (**A**,**B**) Violin plots showing final and maximum counts of monocytes (MONO) and neutrophils (NEU) in patients with pure viral COVID-19 (no bacterial superinfection, *n* = 54) versus those with confirmed bacterial superinfection (*n* = 23). No significant differences were observed. (**C**,**D**) Violin plots showing final and maximum monocyte and neutrophil counts stratified by glucocorticoid (steroid) use (treated, *n* = 34; untreated, *n* = 43). (**E**,**F**) Standardized regression coefficients (β) from multiple linear regression models assessing the independent contribution of qualifying variant (QV) burden in the hemopoiesis and inflammatory response gene sets to final and maximum monocyte and neutrophil counts. Analyses were restricted to pure viral (**E**) and steroid-naïve patients (**F**) and adjusted for COVID-19 outcome. Error bars represent 95% confidence intervals. Significant associations after FDR correction are marked with asterisks.

When comparing patients with pure viral COVID-19 (no bacterial superinfection, *n* = 54 of 77 patients) to those with confirmed bacterial superinfection (*n* = 23), we observed no significant differences in neutrophil or monocyte counts (both final and maximum) between the two groups (Figure 5A,B). The effect of glucocorticoid (steroid) therapy on neutrophil and monocyte counts is shown in Figure 5C,D, where patients were stratified by steroid use (treated, *n* = 34; untreated, *n* = 43). Steroid-treated patients had significantly higher maximum monocyte counts, as well as higher final and maximum neutrophil counts, compared to steroid-naïve patients.

To quantify the independent contribution of genetic burden to monocyte and neutrophil counts, we performed multiple linear regression restricted to pure viral and steroid-naïve patients (*n* = 54 and *n* = 43, respectively). Although not all associations were significant, the relationship between QV burden in the hemopoiesis and inflammatory response gene sets with final and maximum monocyte and neutrophil counts was largely confirmed in the COVID-19 outcome strata (Figure 5E,F) as well as in the disease severity strata (Supplementary Figure S5).

#### 3.5.3. Analysis of Neutrophil- and Lymphocyte-Associated Gene Sets

**Figure 6:**
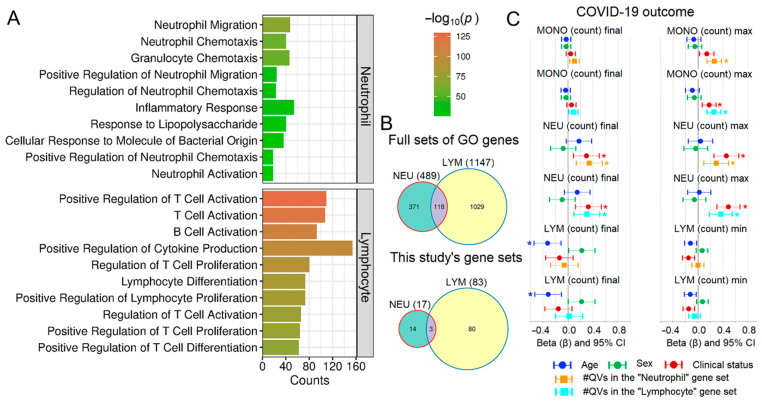
Analysis of neutrophil- and lymphocyte-related gene sets. (**A**) Top 10 GO terms for “neutrophil” and “lymphocyte” search terms. (**B**) Venn diagrams comparing full gene lists with the subset containing QVs. (**C**) Standardized regression coefficients (beta) for the genetic predictor on monocyte, neutrophil, and lymphocyte counts. Asterisks (*) denote significant coefficients after FDR correction.

To further investigate the lack of signal for lymphocyte dynamics, we curated targeted “neutrophil” and “lymphocyte” gene sets using the AmiGO database (Figure 6A,B). While QVs in these cell-specific sets were significantly associated with monocyte and neutrophil counts, as with the broader functional categories, they showed no association with lymphocyte trajectories. (Figure 6C and Supplementary Figure S6). These results reinforce the conclusion that the observed genetic influence is specific to the myeloid lineage.

#### 3.5.4. Internal Stability: Evaluation Model Robustness via Repeated Cross-Validation

**Figure 7:**
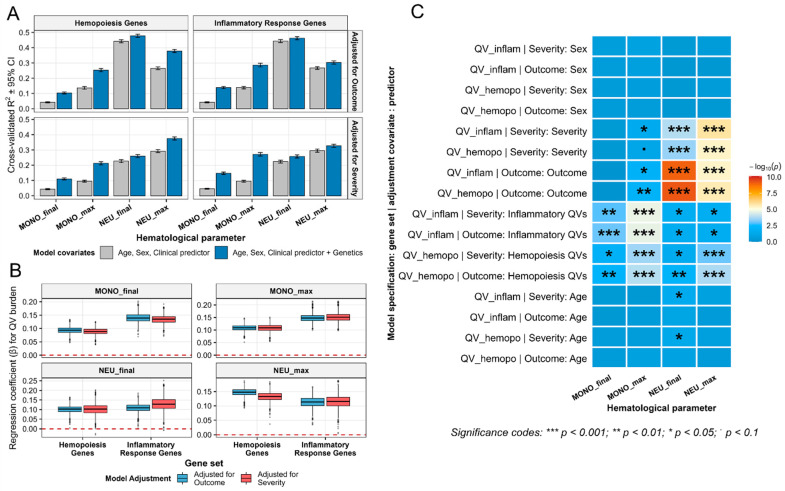
Internal cross-validation confirms robust contribution of rare variant burden to myeloid parameters in severe COVID-19. (**A**) Improvement in cross-validated R^2^ with genetic predictor. (**B**) Stability of the genetic effect across cross-validation resamples. The red dashed line marks β = 0. (**C**) Significance landscape of all model predictors assessed by internal cross-validation. Heatmap of −log_10_ *p*-values for all covariates (genetic, clinical, demographic) in repeated 5-fold cross-validated models predicting monocyte and neutrophil parameters. Asterisks indicate significance levels. All models adjusted for age and sex. MONO—monocytes; NEU—neutrophils; QV—qualifying variants.

An extensive internal validation using repeated 5-fold cross-validation (200 repeats, 1000 resamples) (Figure 7) showed that the addition of the genetic predictor (QV count in inflammatory/hemopoiesis gene sets) consistently increased the cross-validated R^2^ compared to models containing only clinical and demographic covariates (severity/outcome, age, sex). The regression coefficient for the genetic burden remained positive and statistically significant (*p* < 0.05) in all 16 model configurations (2 gene sets × 4 outcomes × 2 adjustment strategies), with the strongest associations for maximum monocyte and neutrophil counts (ΔR^2^ up to +0.177, *p* down to 1.9 × 10^−5^). The most robust signals observed for monocyte parameters (*p* ranging from 1.4 × 10^−4^ to 1.3 × 10^−3^) and maximum neutrophils in hemopoiesis genes (*p* = 1.9 × 10^−4^ and 9.5 × 10^−4^). Age and sex were non-significant in all models, ruling out demographic confounding. Crucially, the cross-validation procedure evaluates model performance on held-out data not used for training, providing an unbiased estimate of predictive ability. The consistency of the genetic signal across 1000 random partitions of the data strongly suggests that our findings are internally robust and not driven by spurious associations or individual outliers.

#### 3.5.5. External Consistency: Evidence from Population-Scale Exome Data and Literature Synthesis

To externally validate our candidate gene sets, we queried the Genebass resource (UK Biobank exome sequencing). Of the 119 genes in our combined hemopoiesis and inflammatory response gene sets, 67 (56.3%) showed nominal association (*p* < 0.05, SKAT-O or Burden test) with neutrophil or monocyte counts or percentages for loss-of-function or missense variants (Supplementary Table S7). This represents an 11.2-fold enrichment over the 5% expected by chance, providing strong independent evidence that our gene sets are enriched for variants that influence myeloid cell abundance in the general population. The goal of this step is enrichment validation, not the discovery of individual genes. The observed 11.2-fold excess of nominally significant genes is statistically robust (binomial test: expected 6, observed 67, *p* = 2.2 × 10^−63^), confirming that the candidate gene sets are not merely a result of noise but are fundamentally linked to the genetic control of the myeloid lineage.

Independent literature review of the 119 genes in our combined hemopoiesis and inflammatory response sets revealed that 75 genes (63%) are supported by strong direct functional evidence linking them to neutrophil or monocyte biology, stress-induced hemopoiesis, or related inflammatory pathways (Supplementary Table S7). Notably, 30 of these functionally validated genes were not captured by Genebass associations, indicating that the candidate gene set is further enriched for myeloid-relevant genes beyond what is detectable by variant burden/SKAT-O tests in the general population. Accordingly, this separate literature-based analysis affirms that the gene sets are not merely statistical aggregates but instead capture genuine functional regulators of myeloid cell behavior in the setting of acute inflammation, supporting their biological validity.

### 3.6. Functional Enrichment and Clinical Trajectories in Phenotypic Extremes

**Figure 8:**
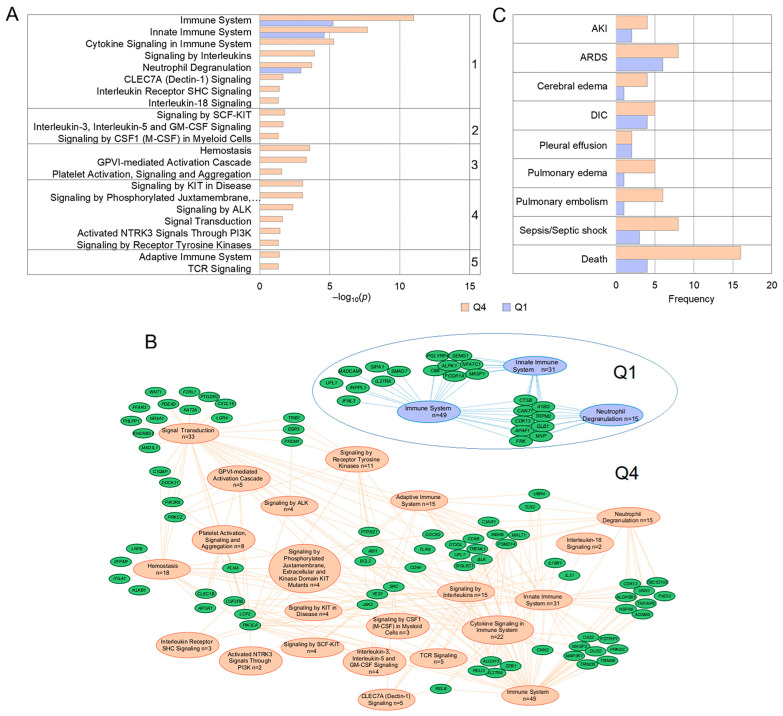
Pathway analysis of genes and clinical complications in phenotypic extremes. (**A**) REACTOME pathway enrichment comparing Q4 (high counts) and Q1 (low counts) groups (FDR *p* < 0.05). Pathways grouped by function: 1-innate immunity; 2-hematopoiesis; 3-hemostasis; 4-RTK signaling; 5-adaptive immunity. (**B**) Gene network visualization. (**C**) Comparison of clinical complications between Q1 and Q4 groups.

The association between rare variant burden and myeloid counts appears highly polygenic (≥90% singleton variants) (Supplementary Table S8). To investigate mechanisms, we performed pathway enrichment on phenotypic extremes, combining high-monocyte/neutrophil (Q4, *n* = 27) and low-count (Q1, *n* = 29) groups. The high-inflammation Q4 group showed broad enrichment (22 pathways) compared to Q1 (3 pathways) (Figure 8A,B; Supplementary Table S9). While both groups shared general immune pathways (e.g., “Neutrophil Degranulation”), Q4 uniquely exhibited pathological signal amplification across multiple systems.

Enriched pathways in Q4 centered on the following: (1) innate immunity/cytokine signaling (e.g., IL-18, CLEC7A); (2) hematopoiesis/myeloid development (SCF-KIT, IL-3/IL-5/GM-CSF, CSF1); (3) hemostasis and platelet function (GPVI, platelet activation); and (4) signal transduction by receptor tyrosine kinases (ALK, NTRK3). This genetic burden translated to severe clinical outcomes (Figure 8C): mortality was four times higher in Q4 (16 vs. 4 deaths; *p* = 0.00065), and vascular events like pulmonary embolism were significantly more frequent (*p* = 0.048). Total complication scores were significantly higher in Q4 (*p* = 0.010), with a composite of thrombo-inflammatory events (PE, Sepsis, DIC, ARDS) showing a near-significant increase (*p* = 0.059), mirroring the pro-thrombotic and hyper-inflammatory genetic profile.

## 4. Discussion

In this study, we integrated longitudinal clinical phenotyping with whole-exome sequencing to unravel the genetic determinants of the dysregulated immune response that characterizes severe and fatal COVID-19. Our analysis revealed that a cumulative burden of rare, predicted protein-disrupting variants within specific hemopoiesis and inflammatory gene sets may serve as a potent driver of myeloid pathology. We demonstrated that this genetic burden significantly predicts the magnitude of neutrophil and monocyte mobilization, which are phenotypes that are centrally implicated in COVID-19 mortality. However, we did not find any association with lymphocyte trajectories. Crucially, this genetic signal remained robust across demographic and clinical subgroups. It persisted in patients without secondary bacterial co-infection and in steroid-naïve patients. This finding suggests that this myeloid expansion is an intrinsic, genetically associated hyperresponsiveness to viral stimuli rather than a reaction to bacterial superinfection or a response to steroid-based therapy. Furthermore, patients with the highest genetic burden exhibited a distinct pathway signature involving innate immune amplification and coagulation defects, which translated clinically into a four-fold increase in mortality and a significantly higher incidence of thrombo-inflammatory complications.

### 4.1. Genetic Impact on Myeloid Dynamics vs. Lymphoid Depletion

An important aspect of our findings is the clear dissociation between myeloid and lymphoid architectures. We found no significant genetic effects on lymphocyte counts. This absence of signal supports the hypothesis that COVID-19-associated lymphopenia is primarily driven by extrinsic, viral-mediated mechanisms, such as direct viral toxicity, trafficking to infected tissues, and cytokine-induced apoptosis [36,37], which likely overwhelm baseline genetic influences. In contrast, the massive expansion of the neutrophil and monocyte compartments represents a ‘production’ response directly constrained by the host’s intrinsic hematopoietic potential. The enrichment of variants in pathways such as SCF-KIT, GM-CSF, and CSF1 implies that the bone marrow ‘set-point’ is genetically tuned. In patients with a high burden of these variants, the immune system appears primed for an exaggerated myeloid output, potentially influencing the upper limits of the inflammatory response.

### 4.2. Functional Relevance of Identified Variants

The variants driving these associations were not merely statistical aggregations but likely functional disruptions. By restricting our analysis to rare “qualifying variants” (QVs) with a median CADD PHRED score of 30.5—placing them in the top 0.1% of deleterious variants in the human genome [38]—we ensured a high probability that these alleles truly disrupt protein function. This rigorous filtering suggests that the observed myeloid dysregulation is associated with a cumulative burden of highly damaging mutations in key regulatory genes, rather than common, low-impact polymorphisms.

### 4.3. Intrinsic vs. Reactive Myeloid Dysregulation: The Impact of Clinical Confounders

A key challenge in interpreting myeloid dysregulation in severe COVID-19 is the potential confounding effect of secondary bacterial infections and life-saving corticosteroid therapy, both of which are potent drivers of leukocytosis. In the present study, we utilized comprehensive electronic health record (EHR) data to distinguish between viral-driven genetic effects and these clinical variables. Our analysis showed that approximately 30% of the cohort developed microbiologically confirmed bacterial superinfection (positive cultures from sputum, BAL, or blood). Interestingly, absolute neutrophil and monocyte counts did not significantly differ between patients with pure viral COVID-19 and those with confirmed secondary bacterial pneumonia. This suggests that the observed myeloid expansion and the associated genetic burden in hemopoiesis-related pathways are primarily characteristic of the host response to SARS-CoV-2 itself, rather than a secondary reaction to bacterial pathogens. This aligns with previous observations that severe COVID-19 triggers “emergency myelopoiesis”—the rapid, premature release of myeloid precursors from the bone marrow—independent of traditional sepsis triggers [10].

Furthermore, we addressed the impact of systemic glucocorticoids, which are known to induce neutrophilia through demargination and to remodel the monocyte compartment [19,20]. While steroid-treated patients in our cohort exhibited significantly higher myeloid counts, our subgroup analysis restricted to steroid-naïve patients confirmed the associations between qualifying variant (QV) burden and myeloid parameters. This indicates that host genetic architecture is a major contributor to the magnitude of myeloid dysregulation, independent of pharmacological interference.

These findings support the idea that the peak neutrophil and monocyte counts reached during a patient’s illness reflect a “genetically governed contributor” of the hematopoietic system’s capacity for emergency myelopoiesis under extreme stress [10]. This is clinically significant because severe COVID-19 outcomes are driven not primarily by direct viral cytopathy, but by a pathologic hyper-inflammatory response that induces tissue damage, vascular leakage, cytokine storm, and thrombosis [39,40,41,42,43]. Our results suggest that the observed myeloid expansion represents a host-related, genetically modulated response to SARS-CoV-2—a form of ‘sterile’ myeloid activation [44].

Consequently, while bacterial superinfection remains a potent driver of late-stage complications and mortality [45,46,47], the genetic underpinnings of myeloid dysregulation appear to be primary. Host genetics shape the initial inflammatory “soil”—a hyper-inflammatory environment that determines the clinical trajectory. This environment likely creates the permissive conditions for secondary infections to take root, rather than being merely a consequence of them. This distinction has profound clinical implications, identifying patients whose sepsis-like hematology is driven by an underlying genetic predisposition. Such patients may benefit more from early, targeted immunomodulation than from the escalation of broad-spectrum antibiotics alone.

### 4.4. Methodological and Biological Context of Genetic Signals

Our genetic analysis revealed two important contextual findings that clarify the nature and detection of the host factors underlying myeloid dysregulation. First, no significant genetic associations were observed for any white blood cell parameter measured at the time of hospital admission. This initial clinical snapshot is heavily influenced by confounding, non-genetic variables, including the precise timing of presentation relative to symptom onset, pre-existing patient comorbidities, age, sex, and immediate interventions [48,49,50]. Such factors introduce statistical noise that can obscure a consistent polygenic signal in a cohort of this size. This result underscores the utility of our longitudinal, peak-count methodology for capturing the host-related, genetically associated potential for emergency myelopoiesis, as detailed in Section 4.3. Second, we found that the cumulative burden of qualifying variants within both neutrophil- and lymphocyte-specific gene sets was significantly associated with peak neutrophil and monocyte counts, but not with lymphocyte trajectories. This observation highlights the extensive pleiotropy and co-regulation inherent to the hematopoietic system. Neutrophils and monocytes share a common granulocyte-monocyte progenitor (GMP), and all major leukocyte lineages are co-regulated by a network of core signaling molecules (e.g., SCF, GM-CSF) [51,52]. Consequently, a high-impact variant in a broadly expressed hematopoietic regulator or even a lineage-specific gene can exert its most statistically detectable and clinically relevant phenotypic effect on the rapidly expanding myeloid compartment, which is central to the pathologic inflammatory response [53,54,55]. This biological crosstalk reinforces our suggestion that the genetic architecture we have identified significantly influences the peak capacity of the myeloid response.

### 4.5. Clinical Consequences: Thrombo-Inflammatory Axes

This genetically determined ceiling of the myeloid response has important clinical implications. Our analysis of phenotypic extremes (Q4 vs. Q1) provides a biological basis for the devastating complications seen in severe COVID-19. Patients with the highest inflammatory cell counts (Q4) carried distinct genetic signatures converging on two pathological axes: (i) A Thrombo-inflammatory Axis: The enrichment of variants in “Platelet Activation” and “Hemostasis” pathways corresponds with the significantly higher rate of pulmonary embolism observed in the Q4 group. This suggests a genetic basis for immunothrombosis, likely mediated by Tissue Factor release from monocytes and NETosis from neutrophils [56,57,58]. (ii) A Systemic Inflammatory Axis: Variants in cytokine signaling and neutrophil degranulation pathways correlated with multi-organ distress and significantly higher mortality [59,60]. This “perfect storm” of genetic factors—a dysregulated hematopoietic system, an immune system primed for cytokine storm, and a coagulation system biased toward thrombosis—may help explain the severe clinical trajectories in the high-burden group.

### 4.6. Strengths and Limitations

First, this study’s primary limitation is its relatively small cohort size (*n* = 77). However, we have mitigated this through several methodological strengths. First, unlike many large-scale GWAS that rely on a single time-point, our study utilized longitudinal hematological data, capturing the dynamic ‘peak’ of the myeloid response. Second, the use of definitive microbiological confirmation and granular steroid treatment records allowed us to rule out major clinical confounders. To address the risk of overfitting and sample heterogeneity, we employed repeated internal cross-validation, which confirmed that the genetic contribution to the myeloid response is stable and reproducible across 1000 random data partitions. Furthermore, the 11.2-fold enrichment of our candidate genes in the population-scale Genebass database provides independent evidence that the pathways identified in our severe COVID-19 cohort are biologically significant. The convergence of results from rare variants in an acute disease setting and rare variants in the UK Biobank suggests a shared genetic architecture governing myeloid homeostasis. While our findings require further replication in larger, multi-ethnic COVID-19 cohorts, the combination of internal stability and external enrichment provides a high degree of confidence in the identified associations.

Second, the study lacks a non-COVID septic control group. Therefore, we cannot definitively determine if this genetic burden is specific to SARS-CoV-2 pathophysiology or represents a general “myeloid priming” that would lead to similar hyper-inflammation in bacterial sepsis or ARDS of other etiologies. Given the fundamental nature of the enriched pathways (e.g., hemopoiesis), we hypothesize that this genotype defines a universal susceptibility to myeloid overreaction under severe physiological stress. This hypothesis is supported by recent transcriptional studies demonstrating that severe COVID-19 and non-COVID-19 sepsis converge after one week in the intensive care unit, with shared patterns of unresolved immune dysfunction and a common set of persistent genes linked to mortality [61,62]. Future studies comparing genetic burden scores between carefully matched COVID-19 and bacterial sepsis cohorts are needed to resolve this important question.

Third, while our study highlights the role of rare host genetic variants, we acknowledge that myeloid dysregulation in COVID-19 is a multi-layered process. Beyond inherited mutations, changes in transcriptional regulation and epigenetic remodeling significantly influence the host response [63,64]. Furthermore, it has been proposed that SARS-CoV-2 RNA may, under specific conditions, be reverse-transcribed and integrated into the host genome [65]. Such integration events could theoretically alter the transcriptional activity of an unknown number of genes, potentially contributing to the immune ‘perfect storm’ observed in severe cases. While our analysis focused on the germline genetic burden, these additional factors—alongside transcriptional noise and viral-induced epigenetic shifts—likely interact with the host’s genetic architecture to shape the final clinical phenotype.

## 5. Conclusions

This study suggests an association between the cumulative burden of rare, high-impact genetic variants in hematopoietic and inflammatory pathways and the magnitude of the myeloid response in severe COVID-19. By integrating definitive microbiological data from electronic health records, we observed that this genetic relationship is identifiable even in patients without secondary bacterial superinfection, supporting the presence of a host-related, ‘sterile’ hyper-inflammatory response to SARS-CoV-2. These associations remained stable across rigorous internal cross-validation and were further reinforced by an 11.2-fold enrichment of these pathways in population-scale exome data from the Genebass resource.

While host genetics represent only one component of the complex multi-layered response to acute viral infection—alongside clinical management and transcriptional regulation—our findings indicate that rare variants may significantly influence the individual capacity for emergency myelopoiesis. These results challenge a uniform approach to severe viral infections, suggesting that genetic stratification could help identify individuals with a predisposition toward a more pronounced myeloid response. For such patients, personalized therapeutic strategies that prioritize early, targeted immunomodulation may be particularly relevant in mitigating the risk of severe complications and mortality. While the absence of a direct replication cohort limits the generalizability of our specific genetic burden score, the consistency of our findings across internal cross-validation, external population-scale exome data, and independent literature evidence supports their biological validity. Direct replication in future large-scale, multi-center COVID-19 studies is warranted to confirm the clinical utility of genetic stratification.

## Data Availability

All raw sequencing data for COVID-19 patients have been submitted to the NCBI BioProject database (https://www.ncbi.nlm.nih.gov/bioproject/ (accessed on 9 September 2025)) under accession number PRJNA947511.

## Supplementary Materials

The following supporting information can be downloaded at https://www.mdpi.com/article/10.3390/v18060604/s1, Table S1: Demographic and clinical characteristics of COVID-19 patients. The data are presented as median and interquartile range (25th–75th percentiles) or *n* (%). Statistical tests used were Fisher’s exact test, the likelihood ratio χ2 test (LR), or the Mann–Whitney U test (MWU) as indicated. Significant results (FDR-adjusted *p*-value) are highlighted in bold. Table S2: Reference ranges and definitions for WBC count endotypes. Table S3: WBC-related measurements in different patient groups. The first block of data (highlighted in grey) includes the first, final, min, and max values for all main subtypes of WBC. The second block of data (highlighted in green) includes the number of hematological analyses per patient in the severe vs. non-severe, and deceased vs. recovered, groups, as well as the day when the maximum monocyte and neutrophil, and minimum lymphocyte, counts in the same groups were registered. Data are given for the full-size groups of patients (severe: *n* = 49, non-severe: *n* = 28; deceased: *n* = 27, recovered: *n* = 50). *p*-values were calculated using the Mann–Whitney U test. Results that remain significant after FDR correction in each block are shown in bold. Table S4: Annotated rare, high-impact variants identified in the 77-patient cohort. Table S5. Full results of the multiple linear regression analysis for monocyte (MONO) and neutrophil (NEU) counts. The table details the MLR analysis for final and maximal cell counts. For each cell type and time point (final/maximal), results are presented in three sequential blocks: (i) Base model; (ii) Base model adjusted for sequencing center; (iii) Base model adjusted for collection clinic (see full model specifications in the note below). Results that remain significant after FDR correction (*p* ≤ 0.0151) are shown in bold. Table S6: WBC-related measurements in different patient groups. *p*-values were calculated using the Mann–Whitney U test. No results remained significant after FDR correction. Table S7: External validation of the myeloid trait associations using the Genebass resource (https://app.genebass.org/) and literature data. References correspond to the main reference list entries [66,67,68,69,70,71,72,73,74,75,76,77,78,79,80,81,82,83,84,85,86,87,88,89,90,91,92,93,94,95,96,97,98,99,100,101,102,103,104,105,106,107,108,109,110,111,112,113,114,115,116,117,118,119,120,121,122,123,124,125,126,127,128,129,130,131,132,133,134,135,136,137,138,139,140,141,142,143,144,145,146,147,148,149,150,151,152]. Table S8: Annotations for genes with QVs within the gene sets of interest. The table indicates which genes are present in patients at the phenotypic extremes (Q4 and Q1) for maximal neutrophil (NEU) and monocyte (MONO) counts. Table S9: REACTOME enrichment analysis results for phenotypic extremes. The table lists results for patient groups with the highest (Q4) and lowest (Q1) neutrophil/monocyte counts. All pathways that were significantly enriched (FDR-adjusted *p* < 0.05) are shown. Figure S1: Gene networks for the GO terms “Immune Response,” “Hematopoiesis,” and “Inflammatory Response”. Figure S2: MLR analysis of genetic effects on basophil and eosinophil counts. This figure shows the percentage of variance (R-squared) explained by the full MLR model for basophil (BASO) and eosinophil (EOS) counts and percentages. The model included non-genetic predictors (age, sex, clinical status) and a genetic predictor (number of QVs per person). The blue line indicates the FDR-corrected significance threshold. Figure S3: Principal Component Analysis (PCA) on the aggregated genetic burden scores (immune response, inflammatory response, hemopoiesis, neutrophil, and lymphocyte gene sets). (A) The PCA colored by sequencing center (Genomed vs. Bio-bank). (B) The exact same PCA colored by recruitment clinic (clinics 1, 2, 3). Figure S4: Demographic subgroup MLR analysis of genetic effects on monocyte and neutrophil counts. The analysis was stratified by sex (males, *n* = 45; females, *n* = 32) and age (≤60 years, *n* = 38; >60 years, *n* = 39). The figure shows standardized regression coefficients (beta) for the genetic predictor from two separate models: one including severity as a covariate (upper panel) and another including outcome (bottom panel). Dummy variables: sex (male 1, female 2); severity (non-severe 0, severe 1); outcome (recovery 0, death 1). Asterisks (*) denote significant coefficients after FDR correction. Figure S5: Clinical subgroup MLR analysis of genetic effects on monocyte and neutrophil counts. Standardized regression coefficients (β) from multiple linear regression models assessing the independent contribution of qualifying variant (QV) burden in the hemopoiesis and inflammatory response gene sets to final and maximum monocyte and neutrophil counts. Analyses were restricted to pure viral (left) and steroid-naïve patients (right) and adjusted for COVID-19 severity. Error bars represent 95% confidence intervals. Significant associations after FDR correction are marked with asterisks. Figure S6: MLR analysis of cell-specific gene set effects, stratified by COVID-19 severity. The analysis was performed to assess the genetic predictor’s effect on monocyte (MONO), neutrophil (NEU), and lymphocyte (LYM) counts. The genetic predictor was the number of QVs in the neutrophil- and lymphocyte-specific gene sets. The figure presents standardized regression coefficients (beta). Covariates in the model included age, sex, and COVID-19 severity. Asterisks (*) denote significant coefficients after FDR correction.
