# Supplementary material for: Genetic Architectures of Myeloid Dysregulation in Severe COVID-19
Source: Viruses. 2026 May 26;18(6):604. doi: 10.3390/v18060604 (PMC13307768; doi:10.3390/v18060604)
Supplement: Supplementary file 1 [file viruses-18-00604-s001.zip › Supplementary Figures.pdf]

Genetic Architectures of Myeloid Dysregulation in Severe COVID-19

Supplementary Materials

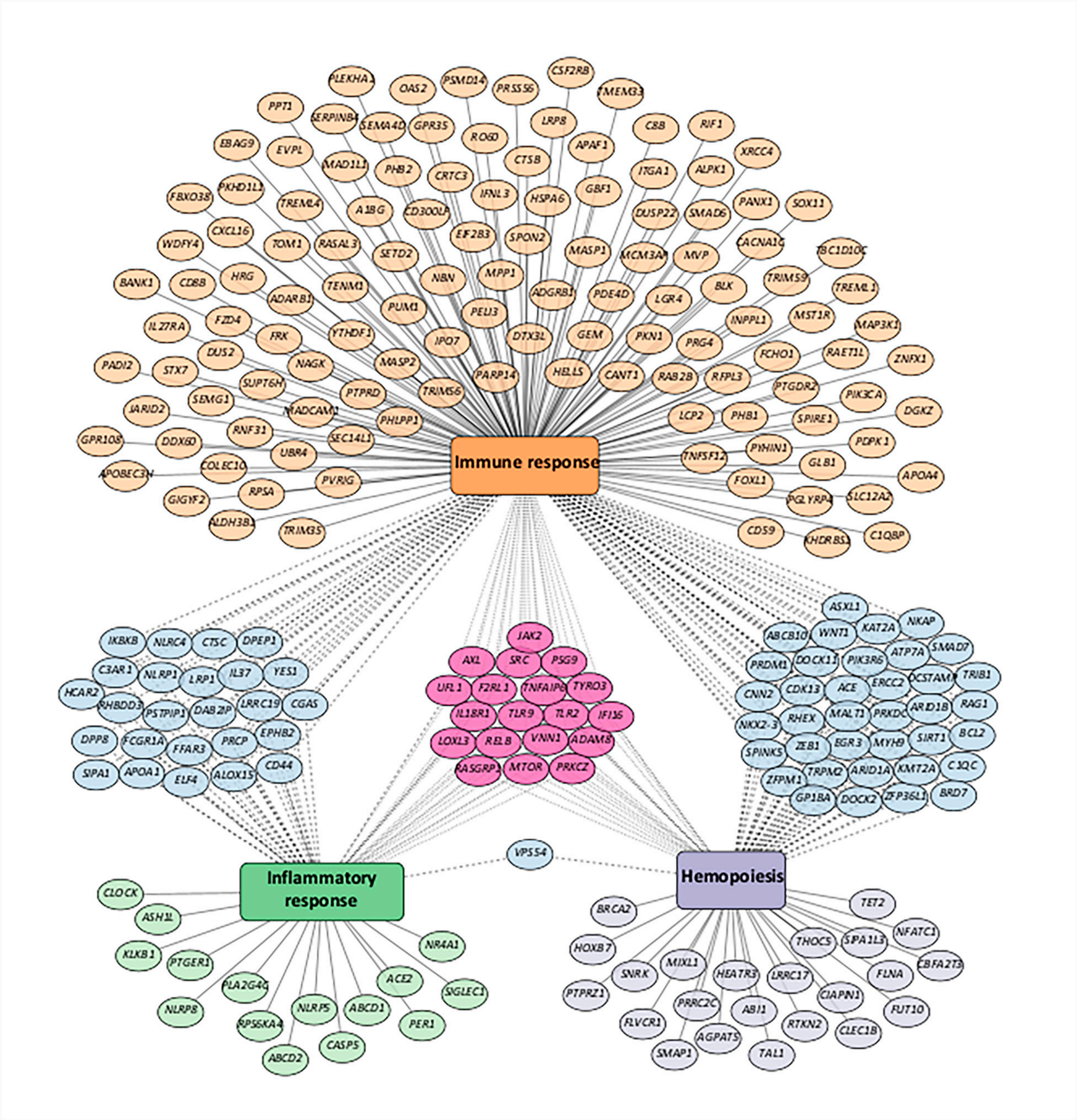

Figure S1: Gene networks for the GO terms "Immune Response," "Hematopoiesis," and "Inflammatory Response".

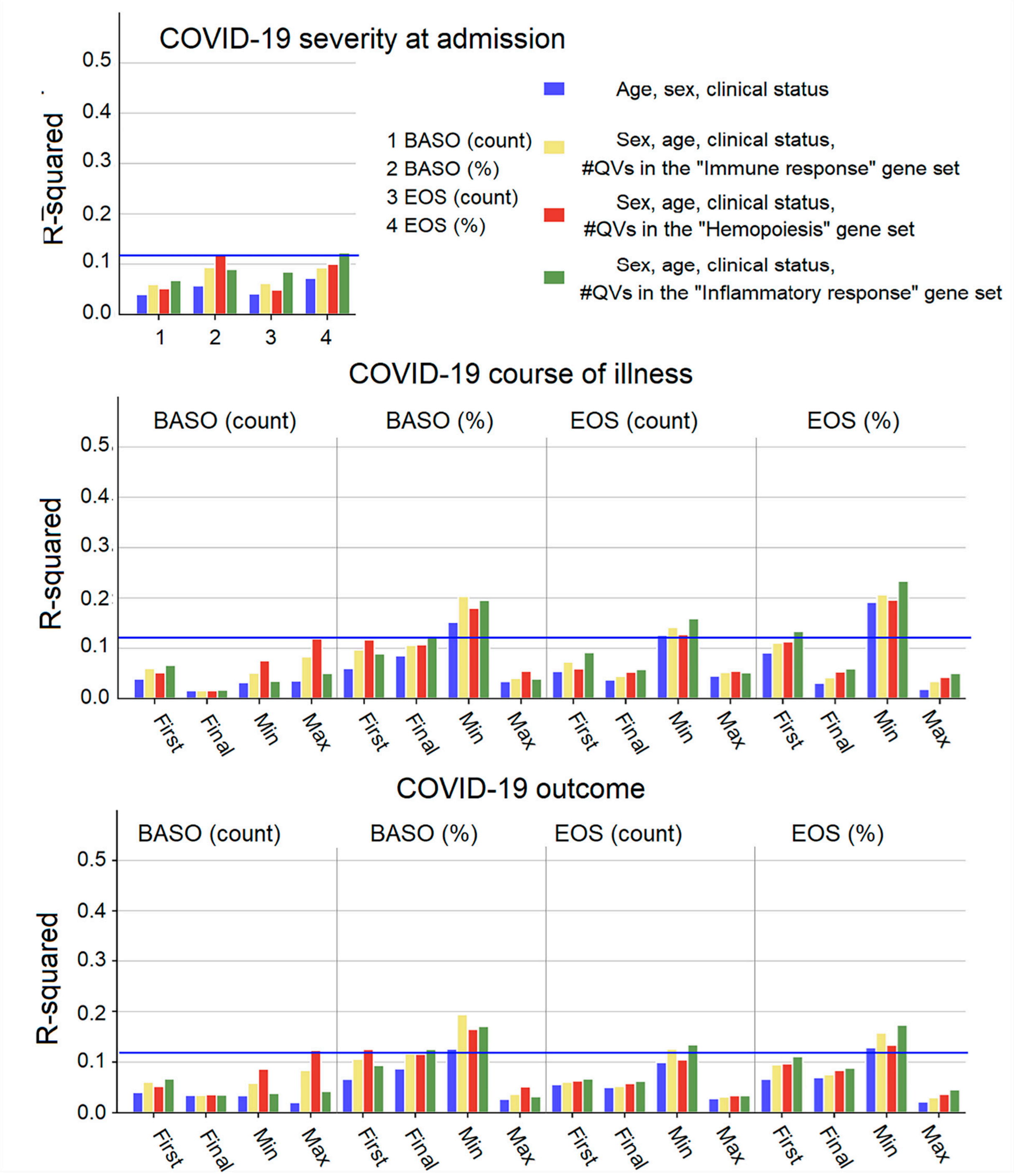

Figure S2: MLR analysis of genetic effects on basophil and eosinophil counts. This figure shows the percentage of variance (R-squared) explained by the full MLR model for basophil (BASO) and eosinophil (EOS) counts and percentages. The model included non-genetic predictors (age, sex, clinical status) and a genetic predictor (number of QVs per person). The blue line indicates the FDR-corrected significance threshold. #QVs indicates the number of qualifying variants within the gene set.

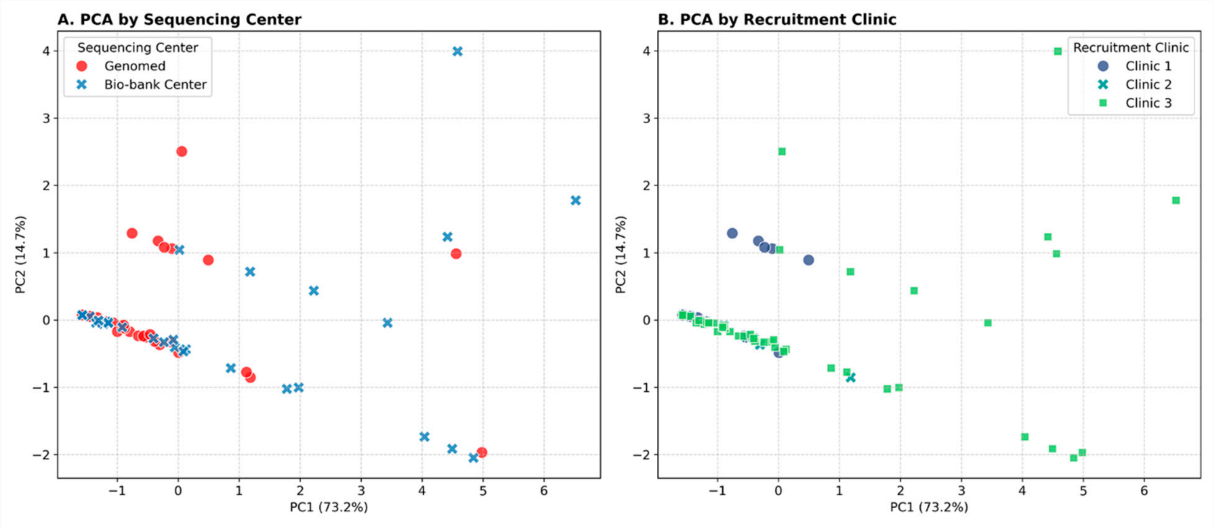

Figure S3: Principal Component Analysis (PCA) on the aggregated genetic burden scores (immune response, inflammatory response, hemopoiesis, neutrophil, and lymphocyte gene sets). (A) The PCA colored by sequencing center (Genomed vs. Bio-bank). (B) The exact same PCA colored by recruitment clinic (clinics 1, 2, 3).

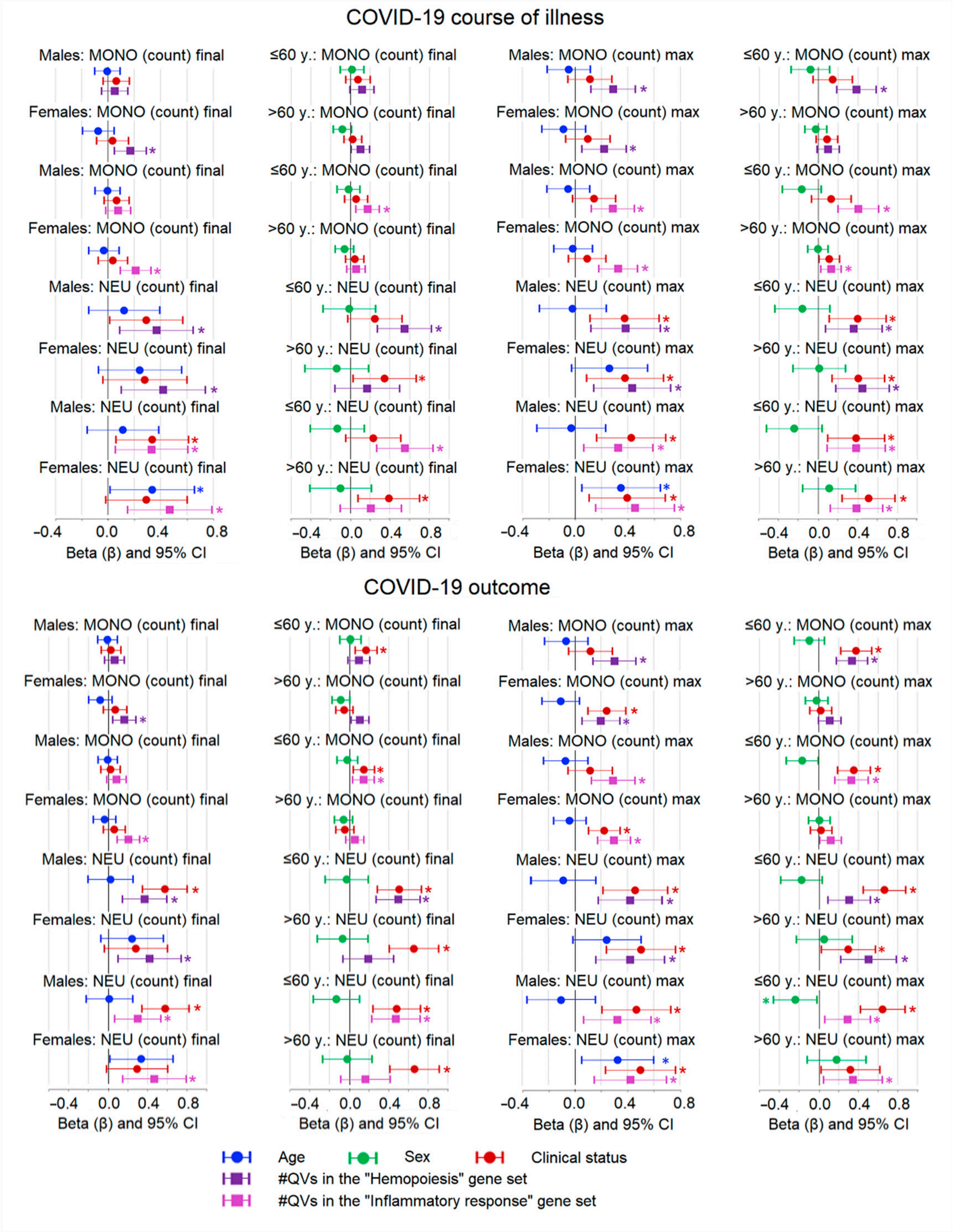

Figure S4: Demographic subgroup MLR analysis of genetic effects on monocyte and neutrophil counts. The analysis was stratified by sex (males, *n* = 45; females, *n* = 32) and age (≤60 years, *n* =3 8; >60 years, *n* = 39). The figure shows standardized regression coefficients (beta) for the genetic predictor from two separate models: one including severity as a covariate (upper panel) and another including outcome (bottom panel). Dummy variables: sex (male 1, female 2); severity (non-severe 0, severe 1); outcome (recovery 0, death 1). Asterisks (*) denote significant coefficients after FDR correction. #QVs indicates the number of qualifying variants within the gene set.

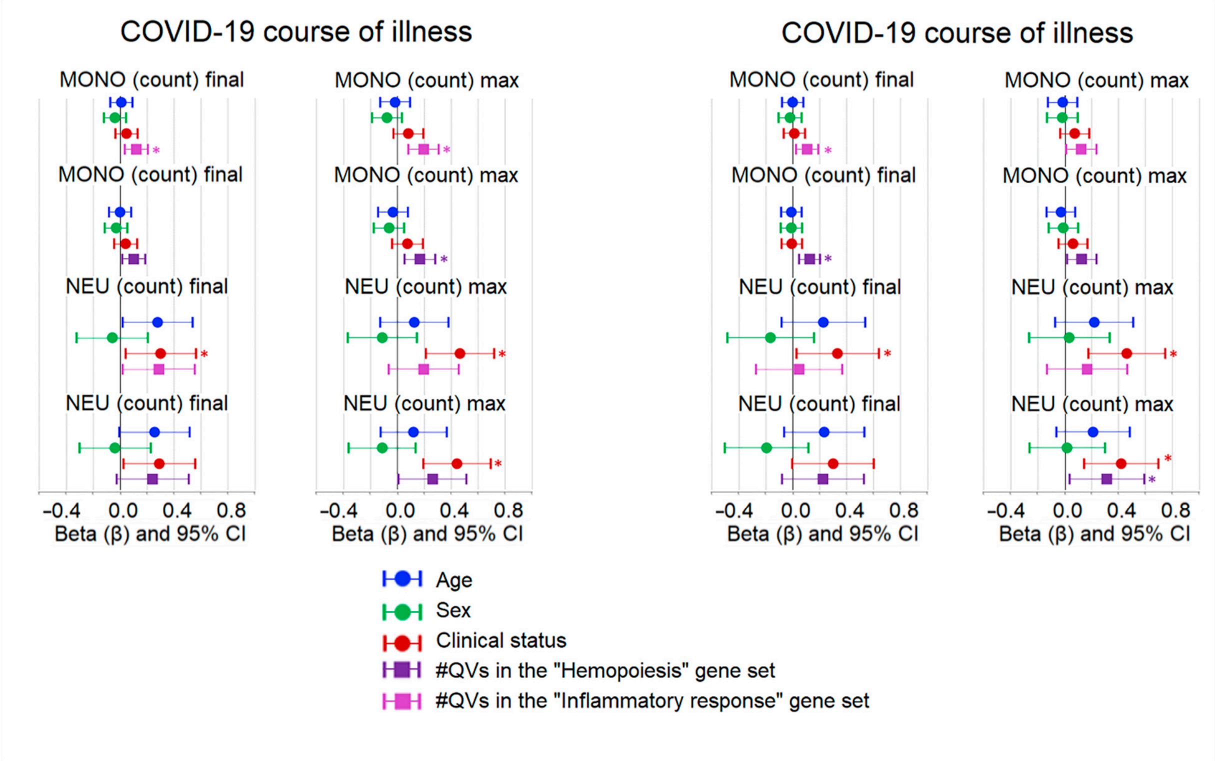

Figure S5: Clinical subgroup MLR analysis of genetic effects on monocyte and neutrophil counts. Standardized regression coefficients (β) from multiple linear regression models assessing the independent contribution of qualifying variant (QV) burden in the hemopoiesis and inflammatory response gene sets to final and maximum monocyte and neutrophil counts. Analyses were restricted to pure viral (left) and steroid-naïve patients (right) and adjusted for COVID-19 severity. Error bars represent 95 % confidence intervals. Significant associations after FDR correction are marked with asterisks. #QVs indicates the number of qualifying variants within the gene set.

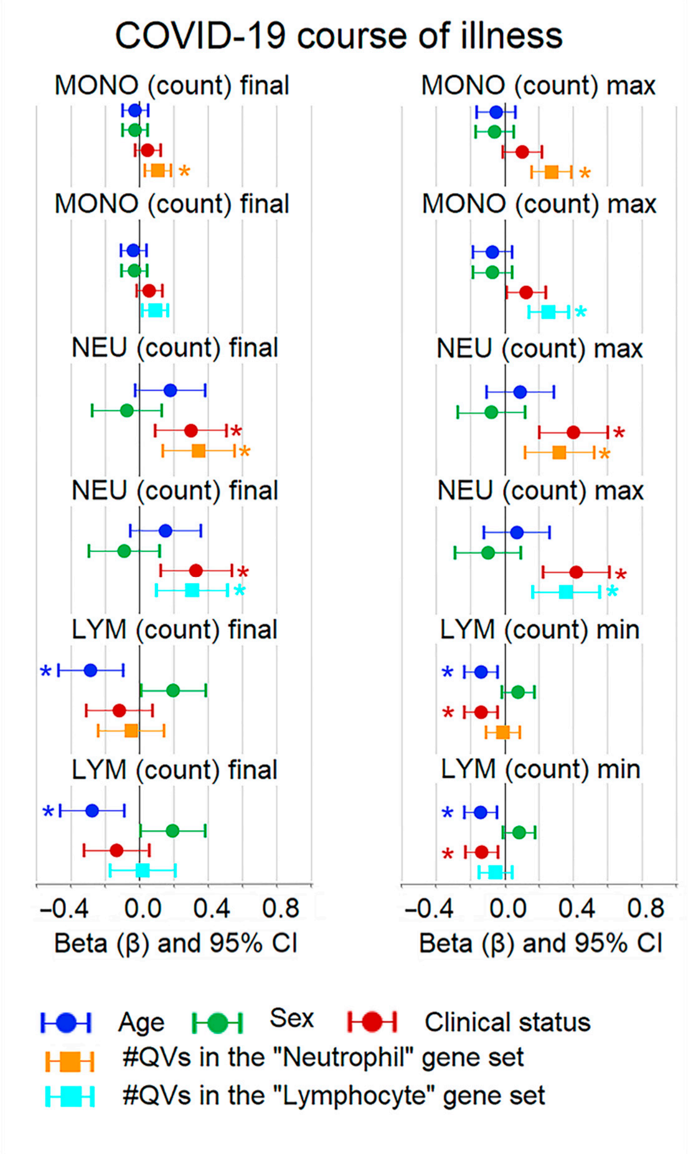

Figure S6: MLR analysis of cell-specific gene set effects, stratified by COVID-19 severity. The analysis was performed to assess the genetic predictor's effect on monocyte (MONO), neutrophil (NEU), and lymphocyte (LYM) counts. The genetic predictor was the number of QVs in the neutrophil- and lymphocyte-specific gene sets. The figure presents standardized regression coefficients (beta). Covariates in the model included age, sex, and COVID-19 severity. Asterisks (*) denote significant coefficients after FDR correction. #QVs indicates the number of qualifying variants within the gene set.
